# A novel irinotecan-lipiodol nanoemulsion for intravascular administration: pharmacokinetics and biodistribution in the normal and tumor bearing rat liver

**DOI:** 10.1080/10717544.2020.1869863

**Published:** 2021-01-27

**Authors:** Marites P. Melancon, Steven Yevich, Rony Avritscher, Adam Swigost, Linfeng Lu, Li Tian, Jossana A. Damasco, Katherine Dixon, Andrea C. Cortes, Nina M. Munoz, Dong Liang, David Liu, Alda L. Tam

**Affiliations:** a Department of Interventional Radiology, Division of Diagnostic Imaging, The University of Texas MD Anderson Cancer Center, Houston, TX, USA; b The University of Texas MD Anderson Cancer Center UT Health Graduate School of Biomedical Sciences, Houston, TX, USA; c Medstar Georgetown University Hospital, Washington Hospital Center, Washington, DC, USA; d Baylor College of Medicine, Houston, TX, USA; e College of Pharmacy and Health Sciences, Texas Southern University, Houston, TX, USA; f The University of British Columbia, Vancouver, Canada

**Keywords:** Nanoparticles, emulsion, drug delivery, irinotecan, lipiodol, pharmacokinetics, biodistribution

## Abstract

Colorectal cancer is one of the most common cancers in the United States and treatment options are limited for patients who develop liver metastases. Several chemotherapeutic regimens have been used for transvascular liver-directed therapy in the treatment of colorectal liver metastases without clear evidence of superiority of one therapy over another. We describe the development of a novel nanoemulsion through combining irinotecan (IRI), a first line systemic agent used for the treatment of colon cancer, with lipiodol, an oily contrast medium derived from poppy seed oil, and evaluated its pharmacokinetic and biodistribution profile as a function of portal venous chemoembolization (PVCE) versus transarterial chemoembolization (TACE) delivery. The Tessari technique was used to create a stable emulsion (20 mg IRI mixed with 2 mL lipiodol) with resultant particle size ranging from 28.9 nm to 56.4 nm. Pharmacokinetic profile established through venous sampling in Buffalo rats demonstrate that the area under the curve (AUC_0−∞_) of IRI was significantly less after PVCE with IRI-lipiodol as compared to IRI alone (131 vs. 316 µg*min/mL, *p*-value = .023), suggesting significantly higher amounts of IRI retention in the liver with the IRI-lipiodol nanoemulsion via first-pass extraction. Subseqent biodistribution studies in tumor-bearing WAG/Rjj rats revealed more IRI present in the tumor following TACE versus PVCE (29.19 ± 12.33 µg/g versus 3.42 ± 1.62; *p*-value = .0033) or IV (29.19 ± 12.33 µg/g versus 1.05 ± 0.47; *p*-value = .0035). The IRI-lipiodol nanoemulsion demonstrated an acceptable hepatotoxicity profile in all routes of administration. In conclusion, the IRI-lipiodol nanoemulsion via TACE showed promise and warrants further investigation as an option for the treatment of metastatic colorectal cancer.

## Introduction

Colorectal cancer is the third most common cancer in men and women in the United States (Cancer.org, 2019). The liver is the most common site of distant metastases and liver metastases are the most common cause of death in patients with colorectal cancer (Wagner et al., 1984; Kemeny, 2007). The treatment of patients with metastatic liver tumors is challenging as surgical resection, the only potentially curative therapy, is an option in less than 30% of patients (Yan et al., 2006). Liver-directed locoregional therapy has improved survival in select patient populations (Park et al., 2014; Vargas et al., 2014). However, a variety of therapies, including chemotherapy delivered via hepatic arterial infusion pumps, selective internal radiation therapy (SIRT), and transarterial chemoembolization (TACE), exist without clear evidence of superiority of one therapy over another.

There are currently two methods widely used in clinical practice to perform TACE. One is based on the conventional approach of mixing an aqueous drug with an iodized oil, such as lipiodol, and creating an emulsion that is delivered intraarterially, followed by embolization with a particulate agent. The other method is based on a one-step TACE using drug-eluting beads loaded with drug. While TACE, with either single agent doxorubicin or doxorubicin used in combination with other chemotherapeutic agents, has been integrated into standard of care treatment options for hepatocellular carcinoma (Llovet et al., 2002), the data supporting TACE for colorectal liver metastases have been less robust. The earlier clinical trials using conventional TACE (drug mixed with lipiodol) employed a variety of chemotherapeutic agents (Sanz-Altamira et al., 1997; Tellez et al., 1998; Leichman et al., 1999; Voigt et al., 2002) but none were able to demonstrate efficacy in survival and only one trial included irinotecan (IRI) (Vogl et al., 2009), which has since become a first-line agent used in the treatment of colon cancer. Vogl et al. treated 463 patients with TACE for colorectal liver metastases either using mitomycin C alone, or mitomycin C in combination with either gemcitabine or IRI but failed to demonstrate a statistical difference between the three treatment protocols (Vogl et al., 2009). The more recent TACE literature focuses on the use of drug-eluting beads as a carrier platform for IRI (DEBIRI) but again, limited data relating to survival outcomes have been documented (Fiorentini et al., 2014; Liu et al., 2015).

IRI, a semisynthetic analogue of the natural alkaloid camptothecin, is a prometabolite that must be hydrolyzed in normal liver parenchyma by the enzymes carboxylesterase 1 and 2 into the active metabolite 7-ethyl-10-hydroxy-camptothecin (SN-38) (Kawato et al., 1991; Guichard et al., 1999; Pommier et al., 2010). SN-38, whose activity is estimated to be 1000 times greater than that of IRI, directly targets the DNA repair enzyme topoisomerase I, which inhibits DNA replication and transcription, promoting apoptotic cell death (Hsiang et al., 1985). As the use of IRI-based first line systemic therapy has been associated with improved survival in metastatic colorectal cancer patients, it is interesting that IRI-based TACE therapy has not been associated with more compelling evidence given that drug concentrations are expected to be higher with direct intra-arterial delivery (Sanz-Altamira et al., 1997; Leichman et al., 1999).

The route of delivery and the carrier platform for IRI are likely to influence its metabolism but how these factors affect the efficiency of drug release, the conversion into SN-38 and its subsequent tumoricidal efficacy are not well studied. Lipiodol, an oily contrast medium derived from poppy seed oil, has known drug delivery and tumor seeking properties (de Baere et al., 1995; Idée & Guiu, 2013), making it a natural candidate as a carrier platform. Despite a historical interest in lipiodol-based chemoembolization, the published literature has failed to establish a role for this technique in the setting of metastatic colorectal cancer (Idée & Guiu, 2013). Rather, drug eluting microsphere therapy (DEBIRI) and SIRT have dominated the published literature as embolization options for the treatment of colorectal liver metastases. Furthermore, liver directed locoregional therapy has largely focused on delivery of therapy via the arterial system as most liver metastases are supplied predominantly by arteries, and to a much lesser extent by portal veins (Bierman et al., 1951; Breedis & Young, 1954; Ridge et al., 1987). The therapeutic efficacy of TACE is based on the premise that the delivery of a chemoembolic agent via the arterial system will result in tumor cell death through ischemia and exposure to higher concentrations of the cytotoxic agent when compared to systemic administration. However, the transarterial route may not be the optimal mechanism for the delivery of IRI: while the prodrug will be delivered directly into the tumor, the exposure of IRI to the normal liver parenchyma is not expected to be significant which may affect the conversion of IRI into its active, tumoricidal metabolite, SN-38.

Transportal venous administration, which exposes both the tumor bearing and normal liver parenchyma to chemotherapy, has been previously reported for adjuvant 5-fluorouracil (5-FU) administration, which showed benefit as a prophylactic neo-adjuvant measure in conjunction with surgical resection but poor curative treatment effect (Archer & Gray, 1990; Laffer & Metzger, 1995; Yu et al., 2012). However, no study has specifically compared the effects of transarterial versus transportal venous administration of single agent IRI for the treatment of colorectal liver metastases. Unlike doxorubicin, the characterization of the resulting drug combination when IRI is mixed with lipiodol is limited. Therefore, we propose to study the pharmacokinetics of IRI mixed with lipiodol in normal rats and its biodistribution when delivered via a transarterial or a transportal venous route in a rat colorectal liver metastases model. We hypothesize that the amount of IRI converted into SN-38 will be greater in the portal venous chemoembolization (PVCE) group compared to the TACE group as drug delivery via the portal vein will result in greater exposure to normal liver parenchyma.

## Materials and methods

All experiments were approved by the institutional animal care and use committee and were performed in accordance with institutional guidelines. GraphPad Prism version 8.0.0 for Windows (GraphPad Software, La Jolla California USA) was used for the calculation of half maximum inhibitory concentration (IC_50_) and statistical analyses outside of the pharmacokinetic calculations.

### Chemicals

The following chemicals were used: Omnipaque 350^®^ (iohexol, 350 mg/mL) nonionic contrast media (GE Healthcare, Chicago, IL), Lipiodol^®^ or ethiodized oil (Guerbet, Princeton, NJ), doxorubicin (DOX) and irinotecan (IRI) (LC Laboratories, Woburn, MA). All other chemicals originated from Sigma-Aldrich, St. Louis, MO and were used without further purification.

### Preparation and characterization of the emulsions

As DOX-lipiodol emulsions have been well described and are routinely used as standard of care regimens during TACE of hepatocellular carcinoma (Llovet et al., 2002; Lammer et al., 2010; Malagari et al., 2010), this drug combination was used as a comparator for the IRI-lipiodol emulsion across measures. Studies have indicated that a water-in-oil emulsion is the optimal configuration for DOX (Llovet et al., 2002; Lammer et al., 2010; Malagari et al., 2010); however, no such data exists for IRI, therefore, both water-in-oil and oil-in-water emulsions (Nastasa et al., 2014) were prepared for *in vitro* characterization. To eliminate processing error, only one person did the optimization procedures. For oil-in water emulsions, 2 mL of iohexol was added to the IRI or DOX (10, 15, or 20 mg dissolved in 2 mL sterile water) solution. This chemotherapy syringe was then connected to a three-way stopcock which was also connected to a syringe containing different volumes of lipiodol (1, 1.5, 2, 4 mL). A homogeneous mixture was obtained by pushing the emulsion through the stopcock in between syringes 20-times (Tessari technique). The Tessari technique is an established emulsification method that produce emulsions that are homogeneous and stable for time intervals for a couple of hours, which is convenient for medical applications (Nastasa et al., 2014).Emulsion droplet diameter was determined using transmission electron microscopy (TEM) and dynamic light scattering (DLS).

TEM was done using JEM1010 transmission electron microscope (TEM; JEOL USA, Inc., Peabody, MA) equipped with a digital camera. Samples for TEM were collected immediately after preparation and added with 2% phosphotungstic acid (PTA) as the staining solution. The pH was adjusted to 7 using potassium hydroxide. The neutral PTA solution was passed through a 0.2 µm filter cartridge prior to use. In a typical staining method, 20 µL of the IRI-lipiodol or DOX-lipiodol sample was adsorbed to the carbon-coated formvar film attached to the copper grid for 1 minute, blotting the excess by touching the edge with filter paper. Immediately, 20 µL of the neutral 2% PTA solution was applied to the grid for 1 minute, blotting the excess again by touching the edge with filter paper. The samples were then allowed to dry before TEM analysis. Image analysis was done to determine the particle size and the size using ImageJ software. The mean diameter and standard deviation were determined from the histogram of the size distribution of the Feret diameter measured from 100 nanoparticles.

DLS, capable of measuring zeta potential, was assessed using ZetaPALS (Malvern Instruments). Every variation of drug dose/lipiodol volume was created three times and for each emulsion, 10 repetitive measurements of mean emulsion droplet diameter were performed at the following time points (*t* = 0, 5, 10, 20, 30, 45, and 60 minutes) and pH settings (PBS for pH = 7.4, saline for pH = 5.4, and Tris buffer for pH = 10.4). To compare the physico-chemical properties of the optimal oil-in-water emulsion, a water-in-oil emulsion, created using the same technique but with the opposite ratio of oil to water was used (20 mg of IRI or DOX in 0.5 mL of sterile water and 0.5 mL of iohexol mixed with 4 mL of lipiodol). ANOVA with Tukey’s post-hoc test was used to evaluate comparisons between groups with *p* < .05 considered to be statistically significant.

### Cell studies and animal models

Buffalo rats (Charles River Laboratories, Wilmington, MA) were used for the experimental studies involving normal liver. The WAG/Rij-CC-531 cells syngeneic model of metastatic colorectal cancer, which has been previously described (White et al., 2016), was used for the experimental studies involving tumor bearing liver. Both the CC-531 cell line, which was generously donated, and the WAG/Rjj rats, which were purchased, originated from the Medical College of Wisconsin.

### Cell studies

The susceptibility of the hepatocyte AML12 cell lines and rat colorectal carcinoma CC-531 to IRI, IRI-lipiodol, blank emulsion (vehicle only), iohexol alone, and lipiodol alone was evaluated in vitro as follows. AML12 cells were grown in a 1:1 mixture of DMEM high glucose/Ham’s F-12 with l-glutamine and supplemented with 10% FBS, 1% Penicillin-Streptomycin, 40 mg/mL dexamethasone, 10 µg/mL insulin, 5 ng/mL sodium selenite and 5.5 µg/mL transferrin. CC-531 cells were grown in DMEM cell culture media containing 10% FBS, 1% Penicillin-Streptomycin and 1% L-Glutamine. Each of these type of cells were plated at a density of 5 × 10^3^ cells/well in 96-well plates and were allowed to attach overnight. The cells were then treated with IRI or IRI-lipiodol, in a range of concentrations from 0.1 to 500 µM for 72 h. Equivalent concentration of iohexol (31.2 mM), lipiodol (21.3 mM), and blank emulsion were added to determine the effect of single agents and vehicle on the survival of the cells. Cells treated with 1% DMSO were used as negative controls. After 72 h post-treatment, the media was replaced to incubate the cells in fresh media containing 10% alamarBlue^TM^. The cell metabolic activity was determined by measuring the fluorescence signal at 590 nm (Cytation5, Biotek). All experiments were done in 5 replicates. The fluorescence intensities produces by the treated cells were expressed as a percentage of that of the untreated cells (control). The IC_50_ values for IRI, IRI-lipiodol, iohexol, and lipiodol were then calculated.

### Interventions

Animals were housed under a standard light-dark scheduled and allowed free access to food and water. For all surgical procedures, animals were anesthetized using 2–3% isoflurane. Orthotopic liver tumors were induced in 23 male WAG/Rjj rats. Using the method as described by White et al. (White et al., 2016) with modifications, the CC-531 cells were suspended to ensure a density of 3 × 10^6^ cells in 0.25 mL of PBS with 2% normal rat serum in preparation for implantation. A 30-gauge needle was used to inject 0.05 mL a suspension of CC-531 cells in to the subcapsular portion of the left lobe of the liver which was exposed via midline incision mini-laparotomy. Hemostatic gauze was applied on the site of implantation to prevent cell reflux and spillage before closing the abdomen. The tumors were then allowed to grow for 2 weeks. T2-weighted 4.7-T magnetic resonance (MR) images were acquired before interventions following the imaging protocol previously described (Munoz et al., 2019) to confirm the location and size of the tumor.

Fifteen normal Buffalo rats (mean weight 337.8 ± 158 g) and 23 WAG/Rjj rats (mean weight 302.5 ± 60.3 g) with minimum tumor diameters of 1 cm by T2-weighted MR imaging received IRI alone or the IRI-lipiodol emulsion via one of three routes of administration: via the tail vein (IV administration), via injection from the portal vein, or via injection from the hepatic artery. Animals who received portal vein drug injection underwent mini-laparotomy to expose and cannulate the portal vein using a 24.5-gauge venous access needle with catheter. A portal venogram was performed with iohexol to identify anatomy. The vascular catheter was gently advanced into the left portal vein and an addition venogram was performed for confirmation. Depending on the assigned treatment group, either IRI or the IRI-lipiodol emulsion was slowly injected under fluoroscopy to minimize reflux from the left portal system. After complete injection, the catheter was removed and hemostasis at the portal vein access site was achieved with prolonged gentle manual compression and a blood stop patch (LifeScience PLUS, Inc. Mountain View, CA).

Animals who received TACE underwent hepatic arterial catheterization using a carotid approach (Nishiofuku et al., 2019). Incision and blunt dissection were used to expose the muscular layer of the left common carotid artery and dissociate the vagus nerve. Using a 20-gauge intravenous catheter (BD Angiocath-IV catheter; 20 G × 1.16”), the carotid artery was cannulated and a 1.6 Fr microcatheter (Tokai Medical, Japan) with a 0.014-inch guidewire (Transcend, Boston Scientific, Natick, MA) were used to select the proper hepatic artery and subsequently the left hepatic artery. Iohexol was injected for digital subtraction angiography and the IRI-lipiodol emulsion was injected under fluoroscopy. Upon completion of the injection of the proposed dose or when stasis of the vessel was observed, the catheters were removed, the common carotid artery was ligated, and the incision closed in two layers with 4–0 Vicryl.

The IRI-lipiodol emulsion used for the interventions was the optimized dose determined from the in vitro studies: an oil-in-water emulsion consisting of 20 mg IRI in 2 mL of sterile water with 2 mL of and 1 mL of lipiodol, giving a concentration of 4 mg/mL. For in vivo studies, all rats received 1 mg of IRI. For PVCE and TACE groups, 1 mg of IRI was dissolved in 100 µL of sterile water and 100 µL of iohexol mixed with 50 µL of lipiodol which is equivalent to 4 mg/mL per rat (20–30 mg/kg rat) (Munoz et al., 2019). In order to compare the pharmacokinetics and biodistribution with IV injection, rats received the same amount of drug (1 mg of IRI), however, the drug was dissolved only in 100 µL sterile water and 100 µL of iohexol.

### Experimental studies

To determine the pharmacokinetics and hepatotoxicity of the IRI-lipiodol emulsion in normal liver, the Buffalo rats were divided into three groups: (1) PV injection with IRI alone (*n* = 4), (2) PVCE with IRI-lipiodol (*n* = 5), and (3) intravenous administration of IRI (*n* = 6 per group). Based on the results of the Buffalo rats, we elected to compare the biodistribution, and hepatotoxicity of the IRI-lipiodol emulsion in WAG/Rjj rats with liver tumors when the emulsion was delivered through the portal vein (PVCE with IRI-lipiodol (*n* = 5) versus intra-arterially (TACE with IRI-lipiodol (*n* = 5)).

Pharmacokinetics was determined by drawing blood samples (0.2 mL) from the Buffalo rats at 0, 5, 15, 30, and 60 min; and 2, 4, 6, 12, and 24 h after injection. Plasma was collected by centrifugation and stored at −20 °C prior to quantitative drug analysis. The concentration of IRI and its active metabolite, SN-38, were determined using high performance liquid chromatography (HPLC) assay. Chromatographic separation was achieved using a reverse-phase of Luna^©^ C18 analytical column (150 × 4.6 mm, 5 µm, Phenomenex) at room temperature. Gradient of two mobile phases, A and B, consist of acetonitrile, and 0.1 M KH_2_PO_4_ buffer containing 3 mM of heptane-sulphonate acid adjusted to pH 4 with 1 M HCl (A = 17:83 v/v and B = 34:66 v/v), was used. The mobile phase was delivered at a flow rate of 1 mL/min with 0–100% mobile phase A during 0–4 min, 100% mobile phase A during 4–17 min, and 100% mobile phase B during 17–22 min. The fluorescence detector wavelengths were set at 380 nm (excitation) and 515 nm (emission) (Rao et al., 2012).

Non-compartmental pharmacokinetic analysis was used to estimate parameters using Phoenix WinNonlin v7.0 software (Pharsight Corporation, Mountain View, CA, USA). Parameters included the area under the plasma concentration – time curve (AUC), mean residence time (MRT), volume of distribution (*Vd*), the plasma clearance (CL) and elimination half-life (*T*_1/2_). The maximum plasma concentration (*C*_max_) were recorded from experimental observations. The AUC_0–_*_t_* and AUC_0–∞_ were estimated by linear up-log-down trapezoidal rule to the last sampling point and extrapolation to time infinity, respectively. Statistical interpretations of the data were performed using Systat 12. Prior to the conduct of any statistical test, the Levene’s test for equality of variances was run on the variances of the observations in the individual groups. If the variances associated with any two mean values were statistically found to be equal or homogeneous, then a parametric test (unpaired Student’s t -test) was used to statistically interpret the mean data. Otherwise, a nonparametric test was employed (Kruskal-Wallis).

To determine the biodistribution of IRI and SN-38 following the administration of the IRI-lipiodol emulsion or IRI alone in the tumor-bearing WAG/Rjj rats were divided into three groups: (1) PVCE with IRI-lipiodol (*n* = 3), (2) TACE with IRI lipiodol (*n* = 3), and (3) IV administration of IRI alone (*n* = 3). Tumor, peritumoral and normal liver were dissected separately as shown in Supplemental Figure 1. Normal liver was collected on the opposite of the tumor-bearing lobe, while the peritumoral area is the liver adjacent to the tumor. Each tissue was mixed with PBS (1:5, v/v) and homogenized prior to HPLC analysis. The HPLC methodology described above was used to quantify the biodistribution of IRI and SN-38. Camptothecin was used as internal standard (final concentration of 100 ng/mL). The mixture was alternately vortexed and sonicated for a total of 3 min, followed by centrifugation at 14,000 g for 3 min. The supernatant was passed through a 0.2 µm filter cartridge and then transferred to an analytical vial for HPLC. The prepared samples were kept in an autosampler at 4 °C until injection (Sai et al., 2002).

Hepatotoxicity in the Buffalo (*n* = 15) and WAG/Rjj rats (TACE with IRI-lipiodol (*n* = 5), PVCE with IRI lipiodol (*n* = 5), IV administration of IRI alone (*n* = 4)) was determined by drawing blood samples and collecting them in tubes with a clot activator gel (BD Microtainer – BD Biosciences NJ) at *t* = 0, 1, 3 and 7 days after injection. Serum was obtained and analyzed for the liver function enzymes alanine transaminase (ALT), aspartate transaminase (AST), alkaline phosphatase (AP) using a Cobas Integra 400 plus analyzer (Roche, Switzerland).

## Results

### Emulsion formulation and characterization

**Figure 1.**
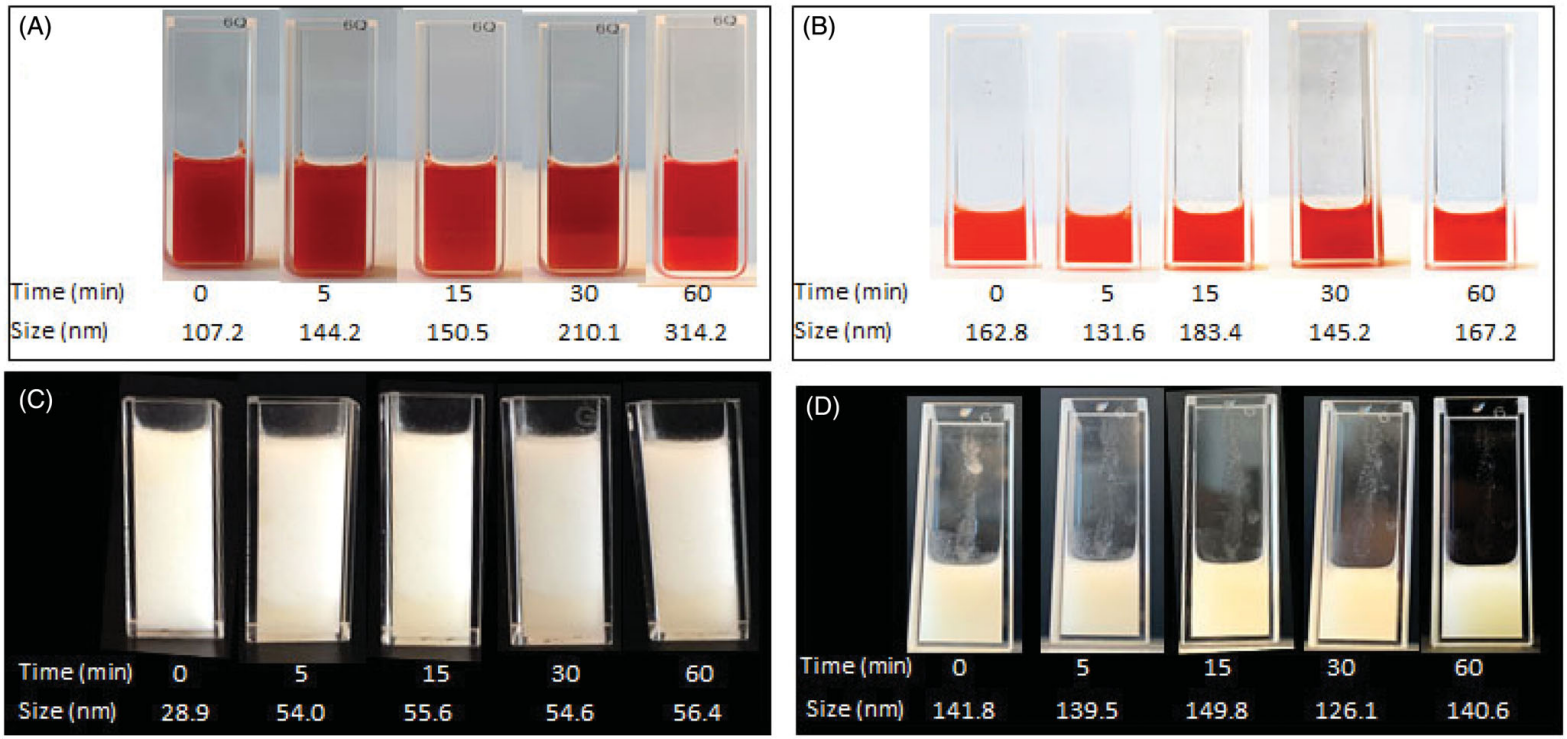
Photographs and sizes of emulsions, prepared using the Tessari method, over time. DOX-lipiodol (A) oil-in-water emulsion and (B) water-in-oil emulsion. IRI-lipiodol (C) oil-in-water emulsion and (D) water-in-oil emulsion.

**Figure 2.**
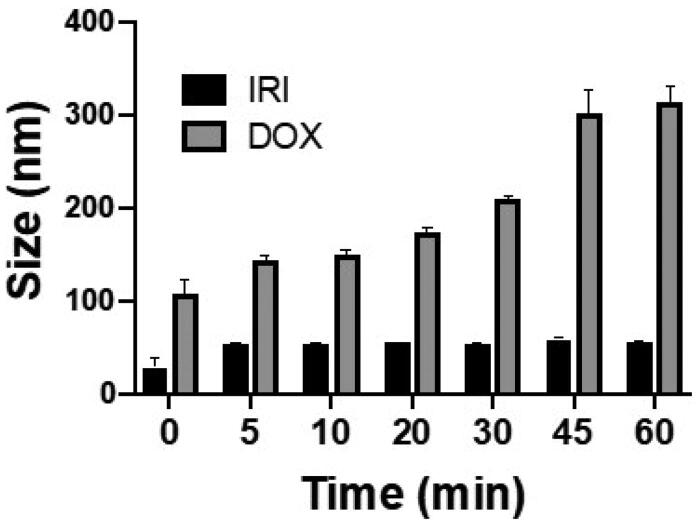
Comparison of the size of the emulsion formed over time between DOX-lipiodol and IRI-lipiodol at the most stable ratio (20 mg drug:2 mL lipiodol).

**Figure 3.**
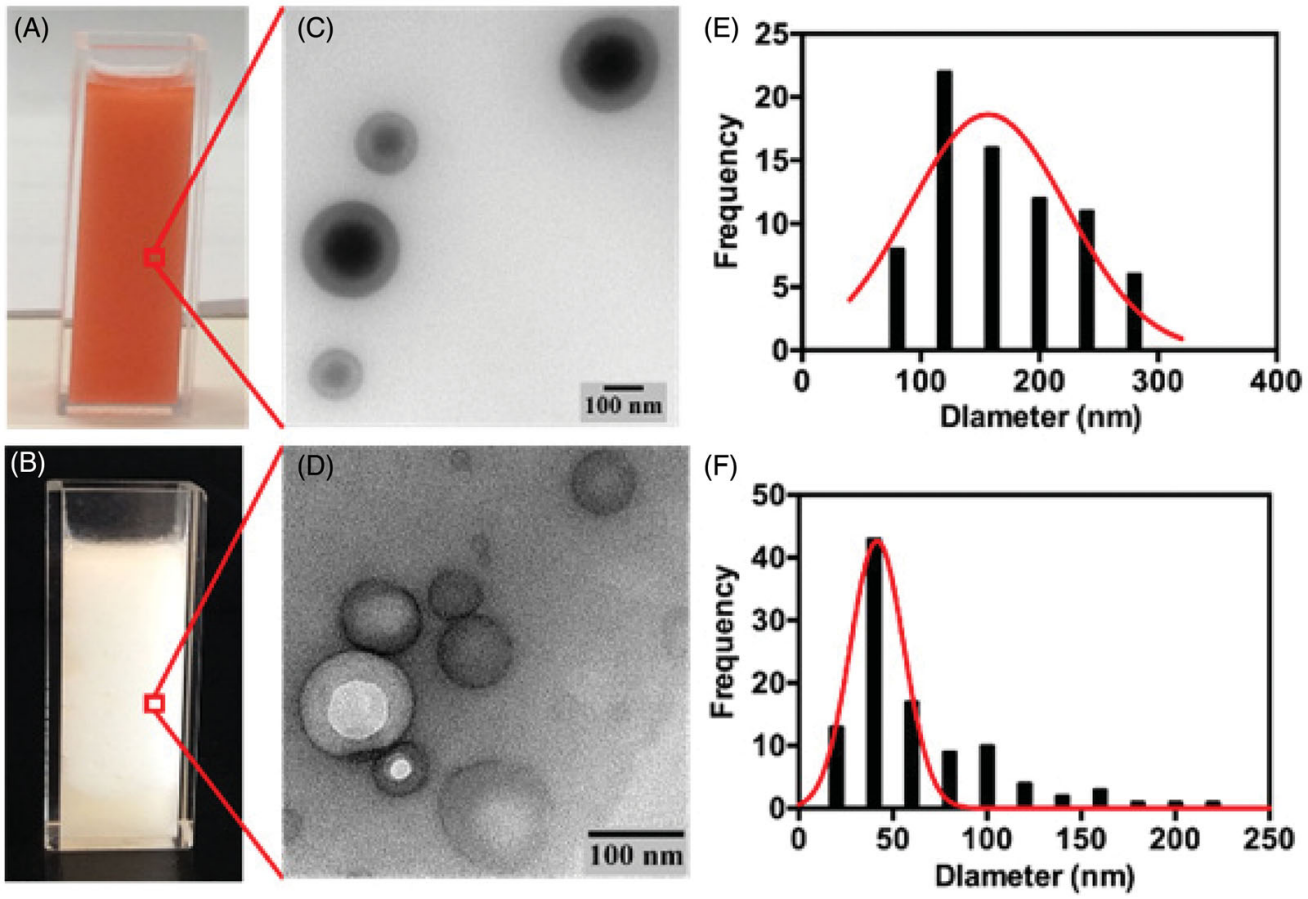
Photographs of DOX-lipiodol (A) and IRI-lipiodol (B) oil-in-water emulsions prepared using the Tessari method with the corresponding transmission electron microscopy images (C,D) and quantification of particle size of the DOX-lipiodol (E) and IRI-lipiodol (F) emulsions.

Figure 1 demonstrates the DOX and IRI emulsions, prepared using the Tessari method, and compares particle sizes of the oil-in-water and water-in-oil emulsions over a period of 1 h. DLS demonstrated the size of the majority of particles in all the emulsions to be in the nanometer range. The particle size of the oil-in-water emulsion of DOX (Figure 1(A)) ranged from 107.2 nm to 314.2 nm, while the particle size of the water-in-oil emulsion (Figure 1(B)) ranged from 162.8 nm to 167.2 nm (*p*-value = .49). For IRI, the particle size of the oil-in-water emulsion (Figure 1(C)) ranged from 28.9 nm to 56.4 nm, which is smaller than the particle size of the water-in-oil emulsion (Figure 1(D)) which ranged from 141.8 nm to 149.8 nm (*p*-value < .0001). Stability was defined as homogeneity of particle size over time and when comparing the different drug-lipiodol concentrations in the emulsions, the most stable formulation and the one which yielded the smallest particle size was 20 mg drug:2 ml lipiodol for both the DOX and IRI emulsions (Supplemental Figure 2). Over the 1- h observation time frame, the particle size of the DOX- lipiodol emulsion ranged from 107.20 ± 16.96 nm to 314.20 ± 16.62 nm and the particle size of the IRI-lipiodol emulsion ranged from 28.93 ± 9.56 to 56.40 ± 0.36 nm (Figure 2). Supplemental Figure 3 illustrates the emulsion particle size and stability for the various drug concentrations (10, 15, 20 mg) and lipiodol volumes (1, 1.5, 2, 4 mL) that were tested. TEM in Figure 3 visually and quantitatively confirmed the particle size of the oil-in-water emulsions of DOX (169.66 ± 59.58) and IRI (62.48 ± 40.98) were consistent with the size determined using DLS.

**Figure 4.**
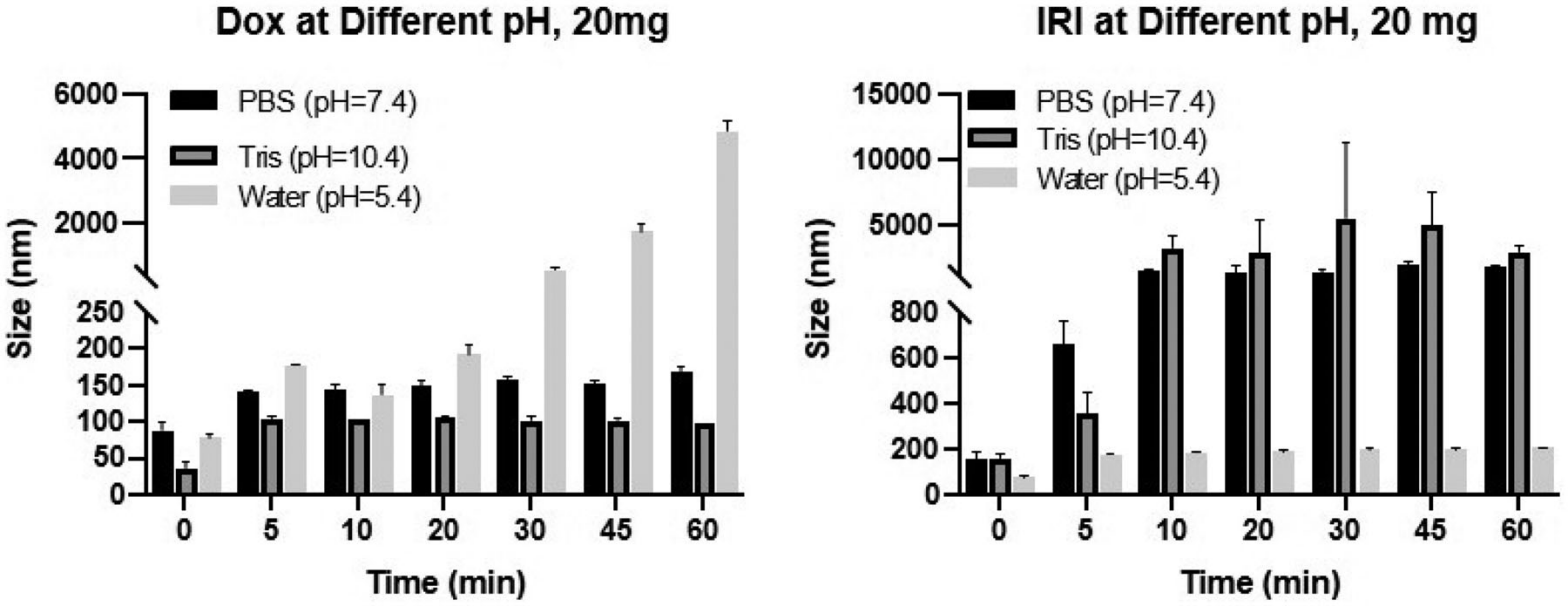
Effect of pH on the particle size of the DOX-lipiodol and IRI-lipiodol emulsions at different pH conditions (20 mg drug:2 mL lipiodol).

We also studied the effect of pH on particle size and stability of the nanoemulsions. The most stable oil-in-water emulsion consisting of 20 mg drug:2 mL lipiodol were tested under three different pH conditions: PBS (pH 7.4), which approximates *in vivo* pH conditions, water (pH 5.4), or Tris buffer (pH 10.4). Figure 4 demonstrates pH conditions effect nanoemulsion particle size and stability. The IRI-lipiodol emulsion was most stable in an acidic environment with particles ranging from 79 nm to 207 nm in size; however, at pH 7.4 and higher, there was a marked increase in size from 159 nm to 661 nm at 5 min, and ultimately to 1891 nm at 1 h. Conversely, the DOX-lipiodol emulsion was most stable at pH 10.4 with the smallest particle sizes over time ranging from 36.6 to 98.5 nm. DOX-lipiodol was also relatively stable in size at pH 7.4 with particles ranging in size from 89 nm to 171 nm over the 1 h period but was least stable in acidic environments, reaching a maximum size of 4889 ± 300 nm.

### *In vitro* cytotoxicity assay of IRI and IRI-lipiodol in CC-531 and AML12 cell lines

**Figure 5.**
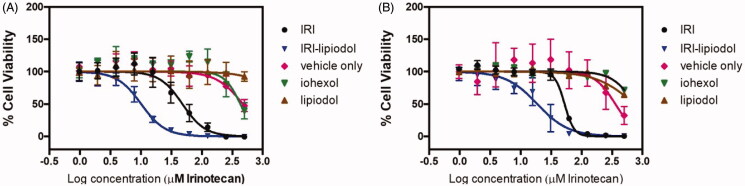
Cytotoxicity of IRI, IRI-lipiodol, blank emulsion (vehicle), iohexol alone, and lipiodol alone against (A) AML12 and (B) CC-531 cell lines. The IC_50_ values of IRI, IRI-lipiodol, iohexol, and lipiodol were 45.23, 10.01, 26,800, and 103,800 µM, respectively, on AML12 cells. For CC-531 cells, the IC_50_ values of IRI, IRI-lipiodol, iohexol, and lipiodol were 55.68, 21.43, 46,600, and 31,300 µM, respectively. Untreated cells were also tested and the viability was 100% for both groups. AlamarBlue^TM^ assay at 72 h was used for all cytotoxicity studies.

The cytotoxicity curves of IRI, IRI-lipiodol, iohexol, lipiodol and blank emulsion (vehicle only) against AML12 and CC-531 cells are shown in Figure 5. In AML12 cells, IRI alone had an IC_50_ value of 45.23 µM, while IRI-lipodol had a lower IC_50_ value of 10.01 µM and was more cytotoxic at higher concentrations than IRI alone (*p* < .0001). Iohexol and lipiodol had an IC_50_ values of 26.8 and 103.8 mM, respectively. This shows that although iohexol and lipiodol have negligible cytotoxicity as that of IRI and IRI-lip, iohexol is more cytotoxic to hepatocytes as compared as lipiodol. Similar trend was observed with CC-531 cells where IRI had a higher IC_50_ value of 52.68 µM compared with IRI-lipiodol of 21.43 µM (*p* < .001), while iohexol and lipiodol had 46.6 and 31.3 mM, respectively. These results show that there are no appreciable toxicity from iohexol and lipiodol at the concentrations used and there is no difference in the cytotoxicity of iohexol and lipiodol for CC531 cancer cells. Furthermore, our results show that IRI and IRI-lipiodol have higher IC50 values in CC-531 (52.56 µM and 21.43 µM, respectively) as compared to AML12 (45.23 µM and 10.01 µM) with *p* < .001.

### Pharmacokinetics, hepatotoxicity, and biodistribution of IRI-Lipiodol nanoemulsion

**Figure 6.**
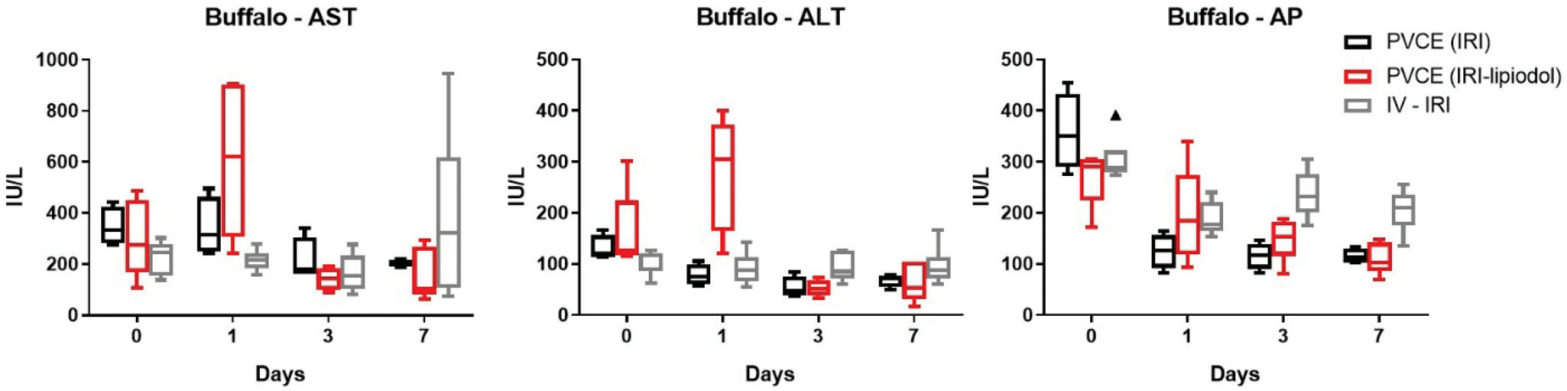
Toxicity data for Buffalo rats after intravenous (IV) or portal vein chemoembolization (PVCE) using irinotecan (IRI) or IRI-lipiodol nanoemulsion (IRI-lipiodol). AST: aspartate aminotransferase; ALT: alanine aminotransferase; AP: alkaline phosphatase.

As the most stable drug formulation, the oil-in-water (20 mg IRI mixed with 2 mL lipiodol) emulsion was used to determine the pharmacokinetic profile of the IRI-lipiodol nanoemulsion administered via the portal vein as compared to the pharmacokinetic profile of plasma concentration of IRI and SN-38 following the administration of the drugs via the portal vein or intravenously. No significant differences of the IRI pharmacokinetic independent parameters (clearance and volume of distribution) were observed among the three groups, i.e., PVCE with IRI, PVCE with IRI-lipiodol, and IV with IRI. Results show that the area under the curve (AUC_0–∞_) of IRI is significantly less after portal vein administration of IRI-lipiodol as injecting IRI alone in normal Buffalo rats. Table 1 presents the calculated parameters of the compared to IRI alone (131 vs. 316 µg*min/mL, *p*- value = .023), suggesting significantly higher amounts of IRI retention in the liver (i.e., first-pass effect) with the IRI-lipiodol nanoemulsion. The mean AUC_0–∞_of SN-38 for the PVEC with IRI- lipiodol group was significantly lower than the IV group (26.1 vs. 121 µg*min/mL, *p* = .006), suggesting a significant portion of SN-38 was localized and not present in the systemic circulation following the portal vein administration of the nanoemulsion. The low AUC may be attributed to the significantly higher clearance of SN-38 in PVCE with IRI-lipiodol group as compared with the IV group (47.1 vs. 12.9 mL/min, *p* = .016). With respect to hepatotoxicity, the PVCE delivery of IRI-lipiodol produced an elevation in both aspartate aminotransferase (AST) and alanine aminotransferase (ALT) enyzmes on day 1; however, the levels of both enzymes returned to baseline by day 3 (Figures 6). In contrast, intravenous or transportal venous administration of IRI alone did not cause major changes in liver biomarkers, AST or ALT. The levels of alkaline phosphatase (AP), on the other hand, decreased in all treatment groups and remained lower than baseline during the follow-up period.

**Table 1.** Summary of pharmacokinetic parameters of irinotean (IRI) and SN-38 in normal Buffalo rats following various interventions.

PK parameters	IRI			SN38		
	PVCE w/ IRI	PVCE w/ IRI-LIP	IV with IRI	PVCE w/ IRI	PVCE w/ IRI-LIP	IV with IRI
*C*_max_ (µg/mL)	1.45 ± 1.17	0.914 ± 0.527	4.42 ± 3.84	0.198 ± 0.094	0.60 ± 0.80	3.06 ± 4.95
AUC_Last_ (µg^.^min/mL)	197 ± 106	119 ± 68.9	144 ± 104	27.7 ± 27.4	21.1 ± 13.9^b^	120 ± 119
AUC_0–∞_ (µg.min/mL)	316 ± 87.6	131 ± 77.5^d^	147 ± 104	41.6 ± 27.2	26.1 ± 13.8^b^	121 ± 119
T_1/2_ (min)	287 ± 130^a^	106 ± 23.4	54.5 ± 14.3	139 ± 46.3^b^	98.8 ± 96.2	41.1 ± 12.6
CL/F (mL/min)	3.44 ± 1.3	10.1 ± 5.78	11.2 ± 7.7	30.2 ± 14.3	47.1 ± 21.4^b^	12.9 ± 6.3
*V_z_*/F (mL)	991 ± 509	1596 ± 1181	863 ± 651	6162 ± 4076^c^	5525 ± 6189	703 ± 344
MRT (min)	296 ± 177^a^	144 ± 28.9^d^	55.7 ± 12.5	217 ± 80	155 ± 144	69.2 ± 23.9

*Note.* IRI: irinotecan; PVCE: portal vein chemoembolization; *C*_max_: maximum concentration; AUC_last_=area under the curve to last measurable concentration; AUC_0–∝_ = area under the curve extrapolated to infinity; *t*_1/2_ = elimination half-life; CL/F = apparent clearance; *V*_z_/F = apparent volume of distribution; MRT = mean resident time.

^a^Statistically significant differences with *p* < .05 between PVCE with IRI and IV.

^b^Statistically significant differences with *p* < .05 between PVCE with IRI-lipiodol and IV.

^c^Statistically significant differences with *p* < .05 between PVCE with IRI and IV.

^d^Statistically significant differences with *p* < .05 between PVCE with IRI and PVCE with IRI-lipiodol.

**Figure 7.**
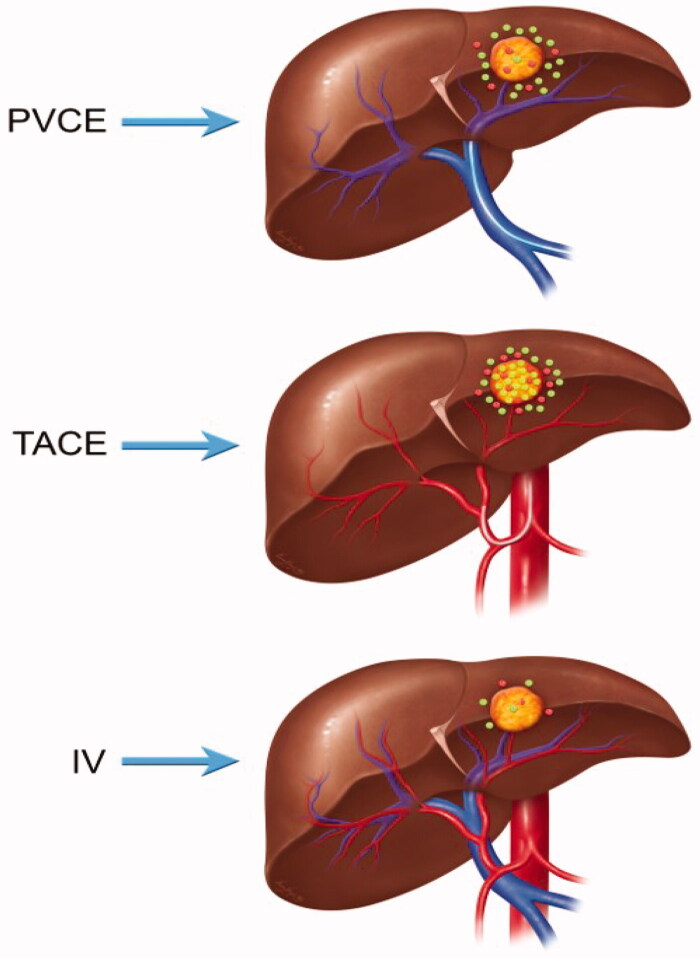
Schematic representation of the different routes of administration and their corresponding biodistribution at 24 h after treatment in tumor-bearing WAG/Rjj rats. Green dots represents IRI, while red dots represents SN38.

Given the acceptable toxicity profile and a distinct pharmacokinetic profile with enhanced liver retention compared to IRI alone, the IRI-lipiodol nanoemulsion was further evaluated in a metastatic liver CRC rat model with the goal of determining the biodistribution when the route of drug administration was varied (Table 2). Biodistribution studies demonstrated that more IRI is present in the tumor following TACE versus PVCE (29.19 ± 12.33 µg/g versus 3.42 ± 1.62; TACE versus PVCE (29.19 ± 12.33 µg/g versus 3.42 ± 1.62; *p*-value = .0033) or IV (29.19 ± 12.33 µg/g versus 1.05 ± 0.47; *p*-value = .0035). Figure 7 shows the schematic representation of the different routes of administration where the green dots represents IRI and the red dots as SN38. In the TACE group, there is more IRI in the tumor as compared to peritumoral and normal liver but the difference between the tumor versus peritumoral and normal liver are not statistically significant (*p*-values = .31). For PVCE, there is less tumor accumulation than the normal liver (*p*-value = .02) and peritumoral area (*p*-value = .30). A similar trend of more IRI accumulation in the normal and peritumoral areas was observed with the IV group compared to the PVCE group (*p*-value = .08) but not statistically significant. Further observation confirmed conversion of IRI to SN-38 but no difference among the groups in terms of SN-38 accumulation in tumor, peritumoral, and normal liver.

**Table 2.** Biodistribution IRI and SN38 in tumor-bearing WAG/Rjj rats at 1 h post-intervention.

Intervention	Area	IRI (µg/g)	SN38 (µg/g)
PVCE	Tumor	3.42 ± 1.62^a^^,c^	1.02 ± 0.55
	Peritumoral	15.73 ± 6.03	3.99 ± 0.93
	Normal	27.83 ± 17.87^c^	5.15 ± 1.86
TACE	Tumor	29.19 ± 12.33^a^^,b^	3.78 ± 2.93
	Peritumoral	13.31 ± 14.73	7.46 ± 9.81
	Normal	11.72 ± 15.22	7.56 ± 11.16
IV	Tumor	1.05 ± 0.47^b^	0.29 ± 0.12
	Peritumoral	3.37 ± 1.48	2.51 ± 0.49
	Normal	3.02 ± 0.72	3.76 ± 0.63

*Note.* IRI: irinotecan; PVCE: portal vein chemoembolization; IV: intravenous; TACE: transarterial chemoembolization.

^a^*p*-Value = .0033 when tumor of PVCE is compared to TACE; ^b^*p*-value = .0035 when tumor of TACE is compared to IV; ^c^*p*-value = .02 when PVCE tumor and normal liver is compared.

**Figure 8.**
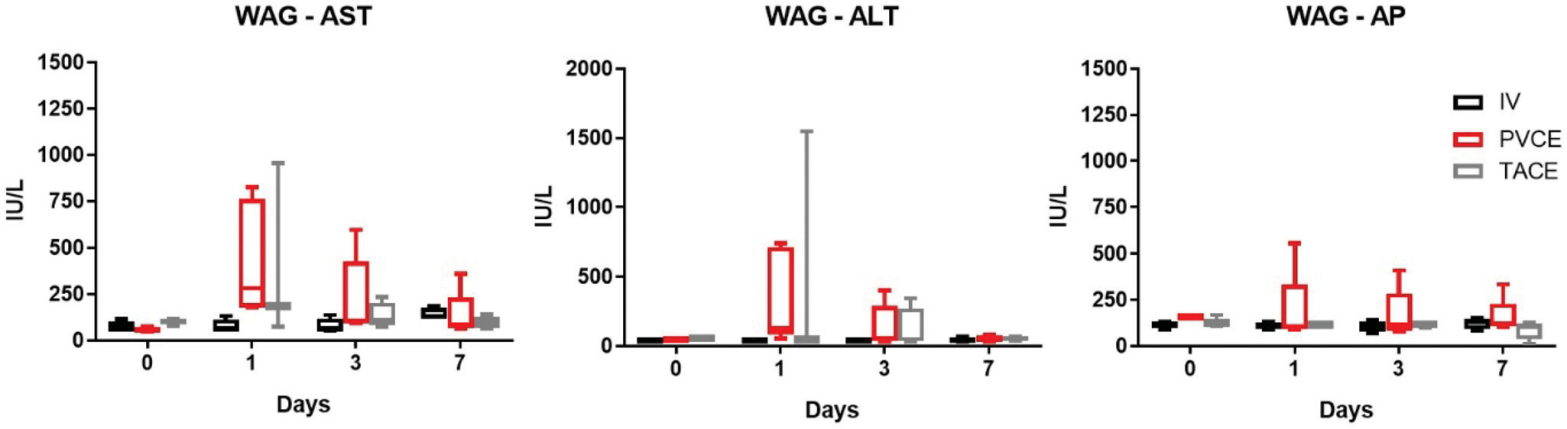
Toxicity data for tumor-bearing WAG/Rjj rats after intravenous (IV) administration of IRI, portal vein chemoembolization (PVCE) or transarterial chemoembolization (TACE) using IRI-lipiodol emulsion (IRI-lipiodol) using the following markers: AST: aspartate aminotransferase; ALT: alanine aminotransferase; AP: alkaline phosphatase (C).

Similar to the hepatotoxicity profile in the Buffalo rats, PVCE was the only treatment that caused a significant increase in both AST and ALT on day 1 in the tumor-bearing WAG rats with enzyme levels gradually declining during the following the days and returning to near baseline values by day 7 (Figure 8). Intra-arterial delivery did not appear to cause significant toxicity in most animals. These findings demonstrate that PVCE was the route of delivery that caused a greater degree, albeit temporary, of hepatic enzyme derangement.

## Discussion

While TACE has become an accepted, standard treatment in the management of patients with hepatocellular carcinoma (Llovet et al., 2002; Lammer et al., 2010; Malagari et al., 2010), chemoembolization treatment options for patients with colorectal cancer liver metastases remain limited and non-standardized (Martin et al., 2011; Fiorentini et al., 2012; Vogl et al., 2018). IRI is one of the most commonly used systemic chemotherapeutic agents in the treatment of unresectable metastatic colorectal cancer but data on its use as a single agent for the endovascular treatment of liver metastases are lacking. In this study, we have successfully fabricated a novel, oil in water, nanoemulsion by combining IRI with the oily carrier, lipiodol, and characterized its *in vitro* and *in vivo* performance characteristics.

Much of the predicate characterization of lipiodol and DOX-lipiodol emulsions was done using light microscopy, in vivo microscopy and viscosity studies (de Baere et al., 1995; de Baere et al., 1996; Cay et al., 1996; Kan et al., 1989, 1997) where the resolution is limited to the micron range. By applying the analysis techniques of DLS and TEM, we were able to discern that the majority of the particles within the DOX- or IRI-lipiodol emulsions were on the nanometer scale, much smaller than previously reported. Many variables can contribute to the stability of an emulsion, including but not limited to the drug being combined, the solvents used for drug reconstitution (He et al., 2018), and the ratio of oil to drug mixture (Kan et al., 1997; Choi et al., 2014). In contrast to the more stable water-in-oil DOX-lipiodol emulsions previously described (Kan et al., 1997), an oil-in-water formulation using a 2:1 IRI:lipiodol ratio resulted in the emulsion that had the smallest particle size and that was the most stable over time. The IRI-lipiodol nanoemulsion was sensitive to pH with the particle size increasing up to 661 nm within 5 min of exposure to a neutral or basic environment. Despite the size increase, the majority of the IRI-lipiodol particles remained under 10 microns in size from 10 to 60 min, well under the reported size ranges of 70–100 microns for DOX-lipiodol particles noted to have the best arterial and portal venous exposure in porcine liver after hepatic artery injection (de Baère et al., 1998) and 20 microns for DOX-lipiodol particles taken up by hypovascular liver metastases in athymic nude mice after intraarterial or intraportal injections (Cay et al., 1996).

The cytotoxic properties of the oil-in-water IRI-lipiodol emulsion, IRI, blank emulsion, lipiodol alone, and iohexol were assessed in normal rat hepatocytes, AML12 cell line, and the CC-531 rat colorectal cancer cell line. Blank emulsion, lipiodol alone and iohexol alone did not have significant cytotoxicity as shown by their IC50 values (Figure 5). This is expected since there has been no report of lipiodol hepatotoxicity, while iohexol is well-tolerated in nephroangiography (Törnquist et al., 1980). Further, only small and transient changes of hepatic enzyme levels following hepatic angiography with iohexol were found in pigs (Jensen et al., 1980). Comparative clinical trials have been performed on various radiologic indications, where iohexol has been compared with conventional ionic contrast media show that there was no significant increase in serum values of hepatic or pancreatic enzymes nor of electrolytes following either of the two contrast media (Billström et al., 1987).

Both IRI and the IRI-lipiodol emulsion were cytotoxic to the AML12 hepatocytes and the CC-531 cells, suggesting that both normal hepatocytes and CC-531 tumor cells are able to convert IRI into the active SN-38 metabolite. The IRI-lipiodol emulsion was more cytotoxic to both groups of cells than IRI alone which may be an effect of lipiodol, which is known to increase the potency of several anticancer drugs by enhancing their intracellular delivery (Towu et al., 2004). Furthermore, both IRI and the IRI-lipiodol emulsion were more cytotoxic to AML12 hepatocytes compared to the CC-531 cells. This may be due to the increased amounts of carboxylesterases, enzymes responsible for the conversion of IRI into its active metabolite SN-38, present in hepatocytes and supports a previous finding indicating that hepatocytes can influence the effect of drugs in vivo by affecting the rate of formation of the hydrolysis products (Williams et al., 1991). The *in vitro* results supported the hypothesis that the amount of IRI converted into SN-38 would be increased with greater exposure of the drug to the normal liver parenchyma as would be achieved with a transportal drug delivery route.

Characterization of pharmacokinetic behaviors of novel dosage formulations and/or route of administrations in normal healthy animals are common to learn how such factors could impact disposition of a drug candidate (Chen et al., 2018; Gao et al., 2019). Several nanoemulsions carrying chemotherapeutics have been shown to improve the bioavailability of the drug and enhance its antitumor efficacy (Seki et al., 2004; Tiwari & Amiji, 2006; Zhao et al., 2013; Alkreathy et al., 2019). Most of these drugs are typically administered orally or intravenously. However, for hepatocellular carcinoma or metastasis in the liver, portal vein and transarterial administration are widely used due to the minimal damage to the liver, longer and higher concentration of chemotherapeutics in the liver (Balogh et al., 2016; European Association for the Study of the Liver; European Organisation for Research and Treatment of Cancer, 2012; Rahbari et al., 2011). Therefore, we evaluated if there was any pharmacokinetic difference of IRI and SN-38 following PVCE versus IV administration of IRI alone. There were no significant differences in the clearance of both IRI and SN-38 via these routes of administration when the IRI was administered as a solution form, suggesting hepatic first-pass metabolism didn’t impact the systemic exposure of IRI and subsequent metabolism to SN-38. However, the PVCE administration of the IRI-lipiodol nanoemulsion did significantly increase the systemic clearance of SN-38 as compared to the IV administration of IRI alone, suggesting a significant hepatic first-pass effect of IRI by nanoemulsion formulation. This provided an indication that PVCE administration of the IRI-lipiodol nanoemulsion might enhance the SN-38 accumulation in the liver. The hepatotoxicity profiles in normal rats and tumor bearing rats were similar, with a transient increase in liver enzymes following embolization with the nanoemulsion that returned to baseline or near baseline levels by day 7.

However, the results of the biodistribution study in the CC-531 WAG/Rjj rat model refuted our hypothesis that the amount of IRI converted into SN-38 would be greater in the PVCE group. Instead, TACE delivery of the IRI-lipiodol nanoemulsion appeared to be superior to PVCE delivery of the IRI-lipiodol nanoemulsion and IV administration of IRI alone with regards to intratumoral deposition of IRI. These findings are in contrast to the findings reported by Cay et al. (1996) where there was no difference in the amount of DOX-lipiodol droplet accumulation within hypovascular tumors as long as the oil droplets were < 20 µm in size (Martin et al., 2011) but consistent with the findings of Kan et al. (1993) who demonstrated that dextran delivered through the arterial system entered the tumor without resistance whereas dextran delivered through the portal vein met resistance at the tumor border with only a small amount entering the tumor itself. Kan et al. (Choi et al., 2014) also demonstrated the terminal portal venules and sinusoids were interrupted at the tumor margins of colorectal liver metastases, contributing to the lack of vascularity within the tumor and representing a potential barrier for drug entry into the tumor via the portal venous route. Our data reveals that the conversion of IRI into SN-38 does occur in this liver tumor model both within tumor tissue and adjacent normal liver. Due to the small number of animals in the study, the known complexity of the tumor microenvironment characterized by heterogeneity in the vascular supply (Nastasa et al., 2014), and the high variability of the data, we were unable to demonstrate significance in the amount of IRI converted into SN-38 in the tumor as a result of the different drug delivery techniques. However, the suggestion that TACE may have an advantage as a route of drug delivery along with evidence that prolonged exposure to SN-38 resulted in superior antitumor activity (Kalra et al., 2014) are valuable to informing future studies that could be designed to evaluate the efficacy of the IRI-lipiodol nanoemulsion.

This reported investigation has several limitations. Variations in the reported data, although addressing the underlying thesis of this exploratory pilot study, may have resulted in statistical under sampling. The animal model was challenging due to the small blood volume of the rat and the technical issues associated with both TACE and PVCE in the species (Bretagnol et al., 2011). Drug stability of the nanoemulsion was likely an issue given the pH results and difficult to accurately assess in vitro drug release. The nanoemulsion may benefit from further optimization of its formulation with the addition of agents to promote drug binding and size stability (Anton et al., 2008; Constantinides et al. 2008; Singh et al., 2017; Chen et al., 2018).

## Conclusion

This initial study evaluated the in vitro and in vivo characteristics of a novel nanoemulsion that was formed by combining IRI with lipiodol. Contrary to previously published results, the study demonstrated that most lipiodol emulsions, whether DOX or IRI, are actually nanometers in size and that oil-in-water or water-in-oil formulations may have specific benefits depending on which chemotherapeutic agents are being combined. The IRI-lipiodol nanoemulsion demonstrated an acceptable hepatotoxicity profile but different pharmacokinetic performance characteristics when compared with the injection of IRI alone, indicating that the addition of lipiodol facilitates the conversion of IRI into SN-38 with better retention in the liver via the first-pass effect. The biodistribution results suggested that the route of administration may be a critical factor for drug delivery. Altogether, the results of this study are promising and indicate that additional studies of the IRI-lipiodol nanoemulsion are warranted to optimize its formulation and evaluate its efficacy for the treatment of metastatic colon cancer. In the future, we plan to translate this study in a pig model, where its anatomy closely resembles that of human. Furthermore, once the pig model showed efficacy, a first in man phase 1 dose escalation trial for determination of tolerability and toxicity in patients with unresectable metastatic colorectal cancer could potentially be done.

## Supplementary Material

Supplemental Material

## References

[CIT0001] Billström Å, Hietala SO, Wirell S. (1987). Effects of metrizoate and iohexol on the liver at visceral angiography. Acta Radiol 28:707–10. 2962603

[CIT0002] Alkreathy HM, Alkhatib MH, Al Musaddi SA, et al. (2019). Enhanced antitumour activity of doxorubicin and simvastatin combination loaded nanoemulsion treatment against a Swiss albino mouse model of Ehrlich ascites carcinoma. Clin Exp Pharmacol Physiol 46:496–505. 30724380 10.1111/1440-1681.13071

[CIT0003] Anton N, Benoit JP, Saulnier P. (2008). Design and production of nanoparticles formulated from nano-emulsion templates-a review. J Control Release 128:185–99. 18374443 10.1016/j.jconrel.2008.02.007

[CIT0004] Archer SG, Gray BN. (1990). Comparison of portal vein chemotherapy with hepatic artery chemotherapy in the treatment of liver micrometastases. Am J Surg 159:325–9. 2305941 10.1016/s0002-9610(05)81228-0

[CIT0005] Balogh J, Victor D, Asham EH, et al. (2016). Hepatocellular carcinoma: a review. J Hepatocell Carcinoma 3:41–53. 27785449 10.2147/JHC.S61146 PMC5063561

[CIT0006] Bierman HR, Byron RL, Kelley KH, et al. (1951). Studies on the blood supply of tumors in man. III. Vascular patterns of the liver by hepatic arteriography in vivo. J Natl Cancer Inst 12:107–31. 14874125

[CIT0007] Breedis C, Young G. (1954). The blood supply of neoplasms in the liver. Am J Pathol 30:969–77. 13197542 PMC1942491

[CIT0008] Bretagnol F, Maggiori L, Zappa M, et al. (2011). Selective portal vein embolization and colorectal liver metastases in rat: a new experimental model for tumor growth study. J Surg Res 171:669–74. 20605581 10.1016/j.jss.2010.03.047

[CIT0009] Cancer.org. Cancer facts & figures. Available at: http://www.cancer.org/research/cancer-facts-statistics/all-cancer-facts-figures/cancer-facts-figures-2019.html [last accessed October 2019].

[CIT0010] Cay O, Kruskal J, Thomas P, et al. (1996). Targeting of Different Ethiodized oil-doxorubicin mixtures to hypovascular hepatic metastases with intraarterial and intraportal injections. J Vasc Interv Radiol 7:409–17. 8761823 10.1016/s1051-0443(96)72880-4

[CIT0011] Chen EM, Quijano AR, Seo Y-E, et al. (2018). Biodegradable PEG-poly(ω-pentadecalactone-co-p-dioxanone) nanoparticles for enhanced and sustained drug delivery to treat brain tumors. Biomaterials 178:193–203. 29936153 10.1016/j.biomaterials.2018.06.024 PMC6082184

[CIT0012] Choi JW, Cho H-J, Park J-H, et al. (2014). Comparison of drug release and pharmacokinetics after transarterial chemoembolization using diverse lipiodol emulsions and drug-eluting beads. PLoS One 9:e115898. 25551760 10.1371/journal.pone.0115898 PMC4281073

[CIT0013] Constantinides PP, Chaubal MV, Shorr R. (2008). Advances in lipid nanodispersions for parenteral drug delivery and targeting. Adv Drug Deliv Rev 60:757–67. 18096269 10.1016/j.addr.2007.10.013

[CIT0014] de Baère T, Denys A, Briquet R, et al. (1998). Modification of arterial and portal hemodynamics after injection of iodized oils and different emulsions of iodized oils in the hepatic artery: an experimental study. J Vasc Interv Radiol 9:305–10. 9540915 10.1016/s1051-0443(98)70273-8

[CIT0015] de Baere T, Dufaux J, Roche A, et al. (1995). Circulatory alterations induced by intra-arterial injection of iodized oil and emulsions of iodized oil and doxorubicin: experimental study. Radiology 194:165–70. 7997545 10.1148/radiology.194.1.7997545

[CIT0016] de Baere T, Zhang X, Aubert B, et al. (1996). Quantification of tumor uptake of iodized oils and emulsions of iodized oils: experimental study. Radiology 201:731–5. 8939223 10.1148/radiology.201.3.8939223

[CIT0017] European Association For The Study Of The Liver; European Organisation For Research And Treatment of Cancer. (2012). EASL-EORTC clinical practice guidelines: management of hepatocellular carcinoma. J Hepatol 56:908–43. 22424438 10.1016/j.jhep.2011.12.001

[CIT0018] Fiorentini G, Aliberti C, Mulazzani L, et al. (2014). Chemoembolization in colorectal liver metastases: the rebirth. Anticancer Res 34:575–84. 24510986

[CIT0019] Fiorentini G, Aliberti C, Tilli M, et al. (2012). Intra-arterial infusion of irinotecan-loaded drug-eluting beads (DEBIRI) versus intravenous therapy (FOLFIRI) for hepatic metastases from colorectal cancer: final results of a phase III study. Anticancer Res 32:1387–95. 22493375

[CIT0020] Gao J, Liu J, Xie F, et al. (2019). Co-delivery of docetaxel and salinomycin to target both breast cancer cells and stem cells by PLGA/TPGS nanoparticles. Int J Nanomedicine 14:9199–216. 32063706 10.2147/IJN.S230376 PMC6884979

[CIT0021] Guichard S, Terret C, Hennebelle I, et al. (1999). CPT-11 converting carboxylesterase and topoisomerase activities in tumour and normal colon and liver tissues. Br J Cancer 80:364–70. 10408839 10.1038/sj.bjc.6690364 PMC2362335

[CIT0022] He M-K, Zou R-H, Wei W, et al. (2018). Comparison of stable and unstable ethiodized oil emulsions for transarterial chemoembolization of hepatocellular carcinoma: results of a single-center double-blind prospective randomized controlled trial. J Vasc Interv Radiol 29:1068–77.e2. 30042075 10.1016/j.jvir.2018.03.027

[CIT0023] Hsiang YH, Hertzberg R, Hecht S, et al. (1985). Camptothecin induces protein-linked DNA breaks via mammalian DNA topoisomerase I. J Biol Chem 260:14873–8. 2997227

[CIT0024] Idée JM, Guiu B. (2013). Use of Lipiodol as a drug-delivery system for transcatheter arterial chemoembolization of hepatocellular carcinoma: a review. Crit Rev Oncol Hematol 88:530–49. 23921081 10.1016/j.critrevonc.2013.07.003

[CIT0025] Jensen LI, Golman K, Almén T, et al. (1980). Effect of angiographic contrast media on the liver. I. Serum concentration of enzymes after coeliacography with balloon catheter in the pig. Acta Radiol Suppl 362:57–63. 6168169

[CIT0026] Kalra AV, Kim J, Klinz SG, et al. (2014). Preclinical activity of nanoliposomal irinotecan is governed by tumor deposition and intratumor prodrug conversion. Cancer Res 74:7003–13. 25273092 10.1158/0008-5472.CAN-14-0572

[CIT0027] Kan Z, Ivancev K, Hägerstrand I, et al. (1989). In vivo microscopy of the liver after injection of Lipiodol into the hepatic artery and portal vein in the rat. Acta Radiol 30:419–25. 2550044

[CIT0028] Kan Z, Ivancev K, Lunderquist A, et al. (1993). In vivo microscopy of hepatic tumors in animal models: a dynamic investigation of blood supply to hepatic metastases. Radiology 187:621–6. 8497606 10.1148/radiology.187.3.8497606

[CIT0029] Kan Z, Wright K, Wallace S. (1997). Ethiodized oil emulsions in hepatic microcirculation: in vivo microscopy in animal models. Acad Radiol 4:275–82. 9110025 10.1016/s1076-6332(97)80029-3

[CIT0030] Kawato Y, Aonuma M, Hirota Y, et al. (1991). Intracellular roles of SN-38, a metabolite of the camptothecin derivative CPT-11, in the antitumor effect of CPT-11. Cancer Res 51:4187–91. 1651156

[CIT0031] Kemeny N. (2007). Presurgical chemotherapy in patients being considered for liver resection. Oncologist 12:825–39. 17673614 10.1634/theoncologist.12-7-825

[CIT0032] Laffer UT, Metzger U. (1995). Intraportal chemotherapy for colorectal hepatic metastases. World J Surg 19:246–51. 7754631 10.1007/BF00308634

[CIT0033] Lammer J, Malagari K, Vogl T, et al. (2010). Prospective randomized study of doxorubicin-eluting-bead embolization in the treatment of hepatocellular carcinoma: results of the PRECISION V study. Cardiovasc Intervent Radiol 33:41–52. 19908093 10.1007/s00270-009-9711-7 PMC2816794

[CIT0034] Leichman CG, Jacobson JR, Modiano M, et al. (1999). Hepatic chemoembolization combined with systemic infusion of 5-fluorouracil and bolus leucovorin for patients with metastatic colorectal carcinoma: a Southwest Oncology Group pilot trial. Cancer 86:775–81. 10463975

[CIT0035] Liu DM, Thakor AS, Baerlocher M, et al. (2015). A review of conventional and drug-eluting chemoembolization in the treatment of colorectal liver metastases: principles and proof. Future Oncol 11:1421–8. 25602287 10.2217/fon.15.3

[CIT0036] Llovet JM, Real MI, Montaña X, et al. (2002). Arterial embolisation or chemoembolisation versus symptomatic treatment in patients with unresectable hepatocellular carcinoma: a randomised controlled trial. Lancet 359:1734–9. 12049862 10.1016/S0140-6736(02)08649-X

[CIT0037] Malagari K, Pomoni M, Kelekis A, et al. (2010). Prospective randomized comparison of chemoembolization with doxorubicin-eluting beads and bland embolization with BeadBlock for hepatocellular carcinoma. Cardiovasc Intervent Radiol 33:541–51. 19937027 10.1007/s00270-009-9750-0

[CIT0038] Martin RCG, Joshi J, Robbins K, et al. (2011). Hepatic intra-arterial injection of drug-eluting bead, irinotecan (DEBIRI) in unresectable colorectal liver metastases refractory to systemic chemotherapy: results of multi-institutional study. Ann Surg Oncol 18:192–8. 20740319 10.1245/s10434-010-1288-5

[CIT0039] Munoz NM, et al. (2019). Comparison of dynamic contrast-enhanced magnetic resonance imaging and contrast-enhanced ultrasound for evaluation of the effects of sorafenib in a rat model of hepatocellular carcinoma. Magn Reson Imaging 57:156–64. 30465870 10.1016/j.mri.2018.11.012

[CIT0040] Nastasa V, Samaras K, Pascu ML, et al. (2014). Moderately stable emulsions produced by a double syringe method. Colloids Surf A 460:321–6.

[CIT0041] Nishiofuku H, et al. (2019). Factors impacting technical success rate of image-guided intra-arterial therapy in rat orthotopic liver tumor model. Am J Transl Res 11:3761–70. 31312386 PMC6614632

[CIT0042] Park J, Chen Y-J, Lu W-P, et al. (2014). The evolution of liver-directed treatments for hepatic colorectal metastases. Oncology (Williston Park) 28:991–1003. 25403632

[CIT0043] Pommier Y, Leo E, Zhang H, et al. (2010). DNA topoisomerases and their poisoning by anticancer and antibacterial drugs. Chem Biol 17:421–33. 20534341 10.1016/j.chembiol.2010.04.012 PMC7316379

[CIT0044] Rahbari NN, Mehrabi A, Mollberg NM, et al. (2011). Hepatocellular carcinoma: current management and perspectives for the future. Ann Surg 253:453–69. 21263310 10.1097/SLA.0b013e31820d944f

[CIT0045] Rao PP, Pascale F, Seck A, et al. (2012). Irinotecan loaded in eluting beads: preclinical assessment in a rabbit VX2 liver tumor model. Cardiovasc Intervent Radiol 35:1448–59. 22358992 10.1007/s00270-012-0343-y

[CIT0046] Ridge JA, Bading JR, Gelbard AS, et al. (1987). Perfusion of colorectal hepatic metastases. Relative distribution of flow from the hepatic artery and portal vein. Cancer 59:1547–53. 3828954 10.1002/1097-0142(19870501)59:9<1547::aid-cncr2820590903>3.0.co;2-6

[CIT0047] Sai K, Kaniwa N, Ozawa S, et al. (2002). An analytical method for irinotecan (CPT-11) and its metabolites using a high-performance liquid chromatography: parallel detection with fluorescence and mass spectrometry. Biomed Chromatogr 16:209–18. 11920947 10.1002/bmc.137

[CIT0048] Sanz-Altamira PM, Spence LD, Huberman MS, et al. (1997). Selective chemoembolization in the management of hepatic metastases in refractory colorectal carcinoma: a phase II trial. Dis Colon Rectum 40:770–5. 9221850 10.1007/BF02055430

[CIT0049] Seki J, Sonoke S, Saheki A, et al. (2004). A nanometer lipid emulsion, lipid nano-sphere (LNS), as a parenteral drug carrier for passive drug targeting . Int J Pharm 273:75–83. 15010132 10.1016/j.ijpharm.2003.12.022

[CIT0050] Singh Y, Meher JG, Raval K, et al. (2017). Nanoemulsion: concepts, development and applications in drug delivery. J Control Release 252:28–49. 28279798 10.1016/j.jconrel.2017.03.008

[CIT0051] Tellez C, Benson AB, Lyster MT, et al. (1998). Phase II trial of chemoembolization for the treatment of metastatic colorectal carcinoma to the liver and review of the literature. Cancer 82:1250–9. 9529016 10.1002/(sici)1097-0142(19980401)82:7<1250::aid-cncr7>3.0.co;2-j

[CIT0052] Tiwari SB, Amiji MM. (2006). Improved oral delivery of paclitaxel following administration in nanoemulsion formulations. J Nanosci Nanotechnol 6:3215–21. 17048539 10.1166/jnn.2006.440

[CIT0053] Törnquist C, Almén T, Golman K, et al. (1980). Proteinuria following nephroangiography. VII. Comparison between ionic monomeric, monoacidic dimeric and non-ionic contrast media in the dog. Acta Radiol Suppl 362:49–52. 6267893

[CIT0054] Towu E, Al-Mufti R, Winslet M. (2004). Uptake of lipiodol-cytotoxics conjugates by hepatocellular carcinoma cells. J Pediatr Surg 39:203–6; discussion 203-6. 14966741 10.1016/j.jpedsurg.2003.10.011

[CIT0055] Vargas GM, Parmar AD, Sheffield KM, et al. (2014). Impact of liver-directed therapy in colorectal cancer liver metastases. J Surg Res 191:42–50. 24990539 10.1016/j.jss.2014.05.070 PMC4134714

[CIT0056] Vogl TJ, Gruber T, Balzer JO, et al. (2009). Repeated transarterial chemoembolization in the treatment of liver metastases of colorectal cancer: prospective study. Radiology 250:281–9. 19092099 10.1148/radiol.2501080295

[CIT0057] Vogl TJ, Lahrsow M, Albrecht MH, et al. (2018). Survival of patients with non-resectable, chemotherapy-resistant colorectal cancer liver metastases undergoing conventional lipiodol-based transarterial chemoembolization (cTACE) palliatively versus neoadjuvantly prior to percutaneous thermal ablation. Eur J Radiol 102:138–45. 29685527 10.1016/j.ejrad.2018.03.015

[CIT0058] Voigt W, Behrmann C, Schlueter A, et al. (2002). A new chemoembolization protocol in refractory liver metastasis of colorectal cancer-a feasibility study. Onkologie 25:158–64. 12006767 10.1159/000055226

[CIT0059] Wagner JS, Adson MA, Van Heerden JA, et al. (1984). The natural history of hepatic metastases from colorectal cancer. A comparison with resective treatment. Ann Surg 199:502–8. 6721600 10.1097/00000658-198405000-00002 PMC1353475

[CIT0060] White SB, Procissi D, Chen J, et al. (2016). Characterization of CC-531 as a rat model of colorectal liver metastases. PloS One 11:e0155334. 27171151 10.1371/journal.pone.0155334 PMC4865145

[CIT0061] Williams FM, Mutch E, Blain PG. (1991). Esterase activity in rat hepatocytes. Biochem Pharmacol 41:527–31. 1997002 10.1016/0006-2952(91)90624-e

[CIT0062] Yan TD, Padang R, Morris DL. (2006). Longterm results and prognostic indicators after cryotherapy and hepatic arterial chemotherapy with or without resection for colorectal liver metastases in 224 patients: longterm survival can be achieved in patients with multiple bilateral liver metastases. J Am Coll Surg 202:100–11. 16377503 10.1016/j.jamcollsurg.2005.08.026

[CIT0063] Yu D-S, Li Y, Huang X-E, et al. (2012). Effect of portal vein chemotherapy on liver metastasis after surgical resection of colorectal cancer. Asian Pac J Cancer Prev 13:4699–701. 23167405 10.7314/apjcp.2012.13.9.4699

[CIT0064] Zhao H, Lu H, Gong T, et al. (2013). Nanoemulsion loaded with lycobetaine-oleic acid ionic complex: physicochemical characteristics, in vitro, in vivo evaluation, and antitumor activity. Int J Nanomed 8:1959–73. 10.2147/IJN.S43892 PMC3666662 23723698

