# Supplementary material for: A novel irinotecan-lipiodol nanoemulsion for intravascular administration: pharmacokinetics and biodistribution in the normal and tumor bearing rat liver
Source: Drug Deliv. 2021 Jan 27;28(1):240–51. doi: 10.1080/10717544.2020.1869863 (PMC8725905; doi:10.1080/10717544.2020.1869863)
Supplement: Supplemental Material [file IDRD_A_1869863_SM8543.pptx]

## Slide 1
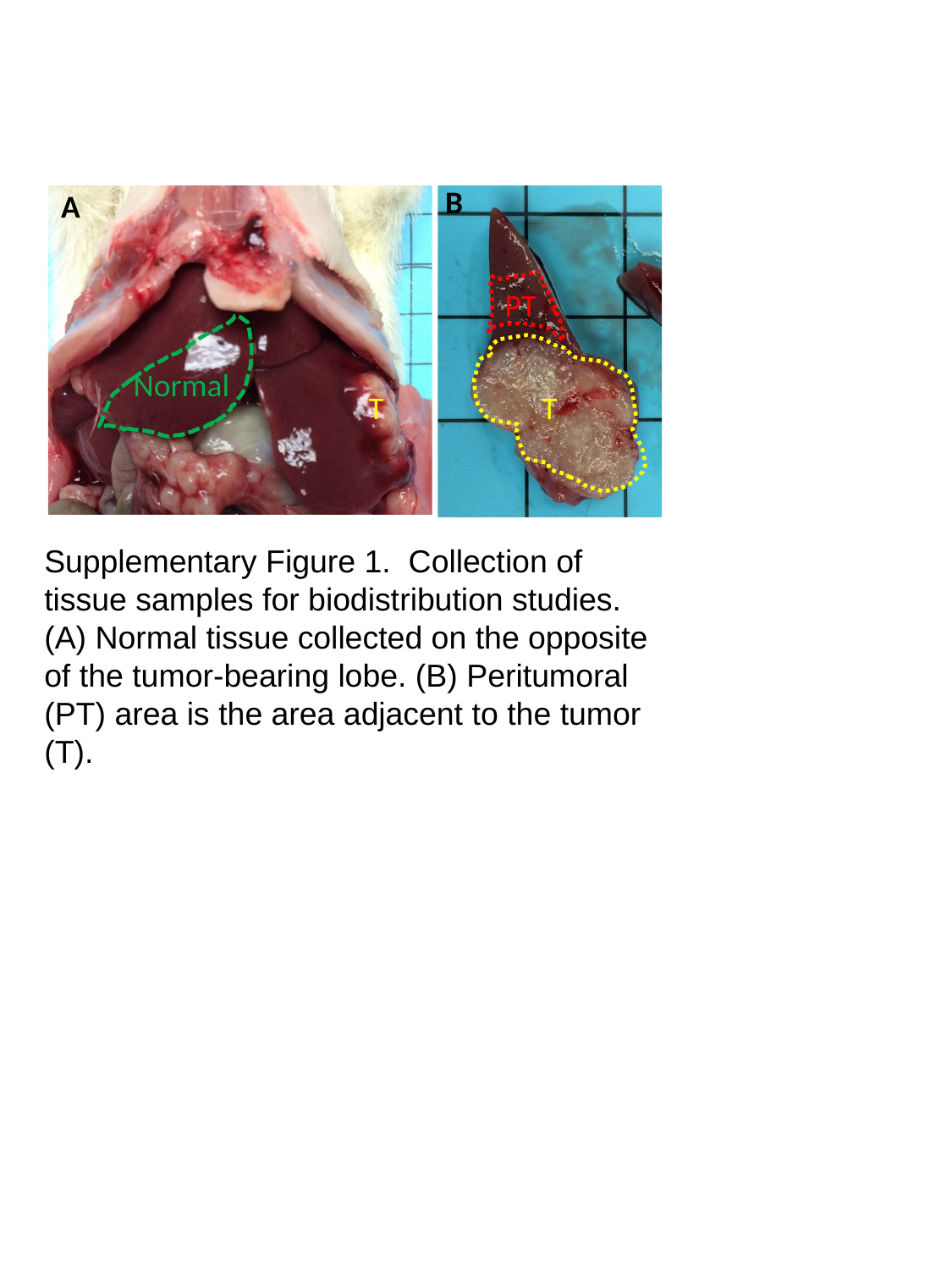

B
A
Normal
PT
T
T
Supplementary Figure 1. Collection of tissue samples for biodistribution studies. (A) Normal tissue collected on the opposite of the tumor-bearing lobe. (B) Peritumoral (PT) area is the area adjacent to the tumor (T).
